# Implementing Physical Activity Studies During COVID-19 and Winter Storms: Lessons Learned

**DOI:** 10.1093/geroni/igab046.811

**Published:** 2021-12-17

**Authors:** Annalisa Na, Calliope Murphy, Tony Chao, Charles Morrison, Karen Chapman, Ronald Lindsey, Mary Hastings

**Affiliations:** 1 Drexel University, College of Nursing and Health Professions, Drexel University College of Nursing and Health Professions, Pennsylvania, United States; 2 University of Texas Medical Branch at Galveston, Galveston, Texas, United States; 3 University Of Texas Medical Branch, Galveston, Texas, United States; 4 Washington University School of Medicine in St. Louis, St. Louis, Missouri, United States

## Abstract

Patient recruitment and retention are challenging for longitudinal studies. Stay-at-home restrictions for the Galveston and Houston regions in 2020 for COVID-19 and in 2021 for the Winter Storms shut down elective healthcare activities and created additional recruitment barriers during the implementation of a 12-month study examining the physical function of older adults receiving a total knee arthroplasty. This presentation describes recruitment and retention strategies during natural disasters. Ten participants started the study during the pandemic and 6 remained through the winter storms (3 withdrew, 1 no showed). Physical activity monitors were distributed and collected through mail, patient reported outcomes were completed online or over the phone, clinician-initiated measures were only collected when clinics were open, and efforts were made to minimize staff burden and follow evolving hospital guidelines. Most importantly, regular communication and follow-up with participants, research team, and department personnel created a sense of community.

